# An explainable machine learning model for predicting preterm birth in pregnant women with gestational diabetes mellitus and hypertensive disorders of pregnancy: development and external validation

**DOI:** 10.3389/fendo.2025.1665935

**Published:** 2025-11-18

**Authors:** Landan Kang, Dan Luo, Wenchi Xie, Xiaojing Luo, Jie Mei, Jing He

**Affiliations:** 1 School of Medicine, University of Electronic Science and Technology of China, Chengdu, Sichuan, China; 2 Department of Obstetrics and Gynecology, Affiliated Hospital of Southwest Medical University, Luzhou, Sichuan, China; 3 Department of Obstetrics and Gynecology, Sichuan Provincial People’s Hospital, University of Electronic Science and Technology of China, Chengdu, Sichuan, China; 4 Department of Nursing, Sichuan Provincial People’s Hospital, University of Electronic Science and Technology of China, Chengdu, Sichuan, China

**Keywords:** preterm birth, gestational diabetes mellitus, hypertensive disorders of pregnancy, Shapley Additive Explanations, Elastic Net regression, risk prediction model

## Abstract

**Background:**

Gestational diabetes mellitus (GDM) and hypertensive disorders of pregnancy (HDP) often coexist and share pathophysiological features such as insulin resistance and endothelial dysfunction, increasing the risk of preterm birth. However, few predictive models have focused specifically on this high-risk group. This study aimed to develop and externally validate a machine learning model for this high-risk population and assess its clinical utility and interpretability.

**Methods:**

This retrospective dual-center study included electronic medical records from 121 and 136 pregnant women with comorbid GDM and HDP, which served as the development and external validation cohorts, respectively. Multiple machine learning algorithms, including Least Absolute Shrinkage and Selection Operator (LASSO) regression, Random Forest (RF), and Naive Bayes (NB), were applied to construct predictive models. To address class imbalance and enhance model robustness, the Synthetic Minority Over-sampling Technique (SMOTE, which generates synthetic samples for the minority class to balance imbalanced datasets) was employed. Model interpretability was further assessed using Shapley Additive Explanations (SHAP).

**Results:**

Thirteen variables with univariate significance were entered into Elastic Net regression, yielding five key predictors: alanine transaminase (ALT), aspartate transaminase (AST), Albumin, lactate dehydrogenase (LDH), and systolic blood pressure at 32 – 36 weeks (SBP_32_36). While the LASSO model achieved the highest area under the receiver operating characteristic curve (AUC, 0.802), the NB model demonstrated greater clinical net benefit, higher reclassification performance as measured by the Net Reclassification Improvement (NRI, which evaluates whether patients are more accurately assigned to higher- or lower-risk groups, which reflects the average improvement in distinguishing high-risk from low-risk patients) and Integrated Discrimination Improvement (IDI), and greater robustness in SMOTE-based sensitivity analyses. In the external validation cohort (n = 136), it maintained strong generalization with an AUC of 0.777 (95% confidence interval [CI]: 0.645–0.887), accuracy of 0.801 (95% CI: 0.735–0.860), sensitivity of 0.792, and specificity of 0.804, supporting its selection as the optimal model for this high-risk population.

**Conclusions:**

The Naive Bayes model exhibited robust predictive ability and interpretability for identifying preterm birth risk in pregnancies with comorbid GDM and HDP, and may serve as a transparent, clinically applicable tool for individualized obstetric risk management.

## 1 Introduction

Gestational diabetes mellitus (GDM) and hypertensive disorders of pregnancy (HDP) are two common pregnancy-related complications that independently increase the risk of adverse maternal and neonatal outcomes, including preterm birth, placental abruption, fetal growth restriction, and perinatal mortality (1). Recent epidemiological evidence suggests that the prevalence of GDM has risen to approximately 14% (2), whereas the prevalence of HDP has increased to around 10% (3). Notably, the incidence of both GDM and HDP has been rising in recent years, with certain studies indicating that the combined prevalence may reach up to 30.4% (4).

This upward trend is partly attributed to increasing maternal age and the implementation of the two-child policy, which have contributed to a growing number of pregnancies affected by both conditions, highlighting the importance of focused perinatal management in this high-risk group (5). Existing studies have identified that factors such as glycemic control levels, mid-pregnancy blood pressure, proteinuria, and a history of preterm birth are closely associated with preterm birth risk (6–9). However, research focusing on the prediction of preterm birth risk in this specific high-risk subgroup of pregnant women with comorbid GDM and HDP remains relatively scarce, with most studies being single-center and small-sample designs (10), lacking external validation, which limits the generalizability and clinical applicability of such models. To date, no prediction models have been specifically developed and externally validated for women with comorbid GDM and HDP.

In addition, although traditional logistic regression models offer good interpretability, they face performance bottlenecks in handling the complex, nonlinear relationships inherent in high-dimensional clinical data (11). In recent years, machine learning algorithms, such as random forest and Extreme Gradient Boosting (XGBoost), have been widely applied in medical prediction studies due to their superior modeling capabilities. Meanwhile, the introduction of interpretability tools such as Shapley Additive Explanations (SHAP) has provided mechanistic explanations for “black-box” models (12), enhancing the clinical interpretability and applicability of these models. By integrating traditional logistic regression and multiple mainstream machine learning algorithms, and systematically evaluating model discrimination, calibration, and clinical utility through receiver operating characteristic (ROC) curves, decision curve analysis (DCA, which evaluates whether using the model provides greater net benefit for clinical decision-making compared with treating all or no patients), and SHAP (which decomposes model predictions to quantify the contribution of each predictor at both the population and individual levels), we aimed to develop an accurate, robust, and interpretable preterm birth risk prediction tool to support early identification and individualized intervention strategies for high-risk pregnancies.

We hypothesized that applying machine learning to GDM–HDP data would yield superior predictive performance for preterm birth compared with traditional models. Therefore, the present study aimed to establish a clinically applicable and interpretable machine learning–based prediction model for preterm birth in women with comorbid GDM and HDP, systematically evaluating its discrimination, calibration, clinical utility, and interpretability.

## 2 Methods

The development cohort included pregnant women who received antenatal care and delivered at Sichuan Provincial People’s Hospital between January 1, 2020, and December 31, 2024, while the external validation cohort included women who delivered at Tongji Hospital, Tongji Medical College, Huazhong University of Science and Technology between January 1, 2022, and December 31, 2023, all of whom met the same inclusion and exclusion criteria. The study was approved by the Ethics Committees of Sichuan Provincial People’s Hospital (No. 2025462) and Tongji Hospital, Tongji Medical College, Huazhong University of Science and Technology (No. TJ-IRB20220611), and all data were anonymized and used solely for research purposes.

The inclusion criteria were as follows. (1) Eligible participants were pregnant women aged over 18 years. (2) All participants met the diagnostic criteria for both GDM and HDP according to the guidelines of the Chinese Society of Obstetrics and Gynecology (CSOG) (13, 14). GDM was diagnosed by a 75-g oral glucose tolerance test (OGTT) performed at 24 – 28 gestational weeks if any of the following plasma glucose thresholds were met: fasting ≥5.1 mmol/L, 1-hour ≥10.0 mmol/L, or 2-hour ≥8.5 mmol/L. HDP was diagnosed as systolic blood pressure (SBP) ≥140 mmHg and/or diastolic blood pressure (DBP) ≥90 mmHg after 20 weeks of gestation, confirmed by at least two measurements taken ≥4 hours apart, or a single measurement of SBP ≥160 mmHg and/or DBP ≥110 mmHg, without subtype differentiation. (3) Participants were required to have received continuous and systematic perinatal management in the hospital from early pregnancy (8 – 15 weeks), with no fewer than five prenatal examinations. (4) Only singleton pregnancies with live births were included.

The exclusion criteria were as follows. (1) Pregnancies complicated by severe chronic systemic diseases, such as systemic lupus erythematosus or malignancies, that could affect pregnancy outcomes were excluded. (2) Pregnancies with major fetal malformations were also excluded. (3) Cases with missing key variables that could not be restored through imputation were excluded.

The primary outcome of interest was preterm birth, defined as delivery occurring prior to 37 gestational weeks (15). Outcome data were obtained from the discharge records, labor course records, and ultrasound information in the electronic medical records system and were independently confirmed by two researchers. Continuous variables were tested for normality using the Shapiro–Wilk test. Normally distributed variables were expressed as mean ± standard deviation (SD), and non-normally distributed variables as median with interquartile range (IQR). Categorical variables were summarized as frequencies (percentages). Group comparisons were performed using the t-test or the Mann-Whitney U test for continuous variables and the Chi-square or Fisher’s exact test for categorical variables, as appropriate. To address missing data, the MissForest algorithm—a non-parametric multiple imputation method based on random forests—was applied. All variables had missing values below 10%, which is generally considered acceptable and unlikely to bias the results. This approach iteratively imputes missing values using regression or classification trees trained on observed data, thereby preserving nonlinear relationships among variables. Ten-fold imputation was conducted separately within the development and validation cohorts to prevent information leakage and maintain dataset integrity.

The candidate predictors encompassed several domains: demographic characteristics (Age, body mass index [BMI]) (16); obstetric history (Adverse Pregnancy History and Primiparity) (17, 18); mode of conception (natural conception or *in vitro* fertilization and embryo transfer [IVF-ET]); pregnancy complications (specifically, the use of antihypertensive medications); and longitudinal measurements of systolic and diastolic blood pressure (SBP and DBP) collected across six gestational intervals: 8 + 0 to 15 + 6, 16 + 0 to 19 + 6, 20 + 0 to 23 + 6, 24 + 0 to 27 + 6, 28 + 0 to 31 + 6, and 32 + 0 to 36 + 6 weeks. Time-specific blood pressure variables were denoted using the format SBP_X_Y or DBP_X_Y, where X_Y indicates the corresponding gestational week range. For example, SBP_32_36 refers to SBP measurements taken between 32 + 0 and 36 + 6 weeks of gestation. Laboratory variables included the mid-pregnancy OGTT (24–28 weeks), with glucose concentrations measured at 0 hours (OGTT-0h), 1 hour (OGTT-1h), and 2 hours (OGTT-2h) after glucose load; liver function markers, including alanine aminotransferase (ALT), aspartate aminotransferase (AST), and lactate dehydrogenase (LDH); as well as Uric Acid, Albumin, and Anemia, totaling more than 30 candidate variables. All variables were collected before the occurrence of outcomes, and outcome data were blinded during data processing to prevent information leakage.

To address the relatively small sample size and the imbalance in outcome distribution, the Synthetic Minority Over-sampling Technique (SMOTE) was applied exclusively to the training folds within cross-validation, while validation and test sets remainedunchanged to avoid information leakage. In this dataset, preterm birth cases represented the minority class, whereas non-preterm cases were the majority class; the minority class was oversampled to achieve a 1:1 ratio with the majority class. We optimized SMOTE’s neighborhood parameter (k) using grid search, selecting k = 5. This value was chosen because it achieved the highest overall and negative class F1 scores (the harmonic mean of precision and recall) during a three-fold cross-validation on the development cohort. This strategy enhanced the model’s sensitivity to preterm prediction while minimizing potential bias introduced by synthetic data. A two-step variable selection process was then implemented. First, univariate logistic regression was conducted to screen candidate predictors, and those with a P-value < 0.20 were retained for further modeling, in accordance with Steyerberg’s recommendation in Clinical Prediction Models to preserve variables with potential predictive value (19). Guided by the events-per-variable (EPV) principle, we aimed to maintain a relatively high EPV value to reduce the risk of overfitting given the limited sample size (development cohort: 121 participants, 31 events). To further address the potential impact of a lower EPV in this context, we applied Elastic Net regularization, combined with three-fold cross-validation, to enhance model stability, and conducted external validation and sensitivity analyses to ensure robustness and generalizability. To reduce multicollinearity and avoid overfitting while maintaining a minimum EPV ratio of at least 10 (20), Elastic Net regression—combining the L1 penalty of Least Absolute Shrinkage and Selection Operator (LASSO) and the L2 penalty of Ridge Regression—was applied to identify the most predictive features. Three-fold cross-validation was used to improve model stability. The final model selection was based on cross-validation performance. It is noteworthy that the use of the Naive Bayes (NB) model was pre-specified in our analysis plan, given its advantages in small-sample scenarios and its probabilistic interpretability. It was not chosen *post-hoc* based on its performance on an external validation set. A total of five key predictors were ultimately retained for final model development.

Model performance was evaluated across multiple dimensions: (1) discrimination was assessed using ROC curves and area under the curve (AUC) values; (2) calibration was evaluated using the Hosmer-Lemeshow test and calibration plots to assess agreement between predicted probabilities and observed outcomes; (3) clinical utility was examined using DCA to estimate net benefit under different threshold probabilities; (4) interpretability was evaluated using SHAP to quantify the direction and contribution of each predictor to individual predictions; (5) generalizability was assessed using an external validation cohort; and (6) reclassification performance was evaluated using integrated discrimination improvement (IDI) and net reclassification improvement (NRI) indices. All statistical analyses were conducted using R (version 4.2.3) and Python (version 3.12), with a two-sided P-value < 0.05 considered statistically significant.

## 3 Results

### 3.1 Characteristics of participants

A total of 257 pregnant women diagnosed with GDM and HDP were included in this study. The development cohort comprised 121 cases from Sichuan Provincial People’s Hospital, among whom 31 (25.62%) experienced preterm birth and 90 (74.38%) had non-preterm birth. The external validation cohort included 136 cases from Tongji Hospital, Tongji Medical College, Huazhong University of Science and Technology, among whom 24 (17.65%) experienced preterm birth and 112 (82.35%) had non-preterm birth. Baseline characteristics were compared between the development and validation cohorts to evaluate their population comparability. Significant differences were observed in several key variables, including ALT, AST, Total Bilirubin, and DBP across multiple gestational weeks (8–31 weeks). All P-values were less than 0.001.

Additionally, the incidence rates of History of HDP, Medication, Cardiovascular Disease, Anemia, Twin Pregnancy, FPG_32_36, and IVF-ET differed significantly between the two cohorts. These discrepancies might be attributable to variations in clinical management practices or differences in population characteristics between the two centers. However, no statistically significant differences were found in Age, BMI, OGTT results, Albumin, Creatinine, or most Weight measurements. Importantly, the proportion of preterm births did not differ significantly between the two groups (P = 0.161), indicating general comparability in the outcome of interest, as detailed in **Table 1**.

**Table 1:** Baseline characteristics of the development and validation cohorts.

Variable	A (n=121)	B (n=136)	P
Age (years)	32.21 ± 4.51	32.85 ± 3.87	0.231
BMI (kg/m²)	25.00 (22.70 - 27.70)	23.95 (22.00 - 26.62)	0.107
Adverse Pregnancy History	9 (7.44%)	10 (7.35%)	1.000
Primiparity	80 (66.12%)	106 (77.94%)	0.048
IVF_ET	10 (8.26%)	24 (17.65%)	0.042
Medication	57 (47.11%)	10 (7.35%)	<0.001
SBP_8_15 (mmHg)	126.00 (118.00 - 134.00)	128.00 (124.00 - 131.25)	0.055
SBP_16_19 (mmHg)	126.00 (116.00 - 134.00)	126.00 (122.00 - 130.00)	0.696
SBP_20_23 (mmHg)	127.36 ± 11.84	129.00 (124.00 - 135.00)	0.172
SBP_24_27 (mmHg)	127.85 ± 9.97	129.00 (126.00 - 134.00)	0.082
SBP_28_31 (mmHg)	128.60 ± 11.73	129.64 ± 11.23	0.472
SBP_32_36 (mmHg)	136.82 ± 12.68	134.17 ± 14.72	0.127
Preterm Birth	31 (25.62%)	24 (17.65%)	0.161
DBP_8_15 (mmHg)	80.00 (72.00 - 85.00)	86.00 (81.00 - 90.00)	<0.001
DBP_16_19 (mmHg)	76.69 ± 10.73	82.00 (79.00 - 87.25)	<0.001
DBP_20_23 (mmHg)	77.50 ± 10.02	82.00 (79.00 - 88.00)	<0.001
DBP_24_27 (mmHg)	79.07 ± 9.95	83.00 (80.00 - 87.25)	<0.001
DBP_28_31 (mmHg)	80.17 ± 9.35	84.97 ± 8.89	<0.001
DBP_32_36 (mmHg)	85.00 (78.00 - 92.00)	86.00 (81.00 - 92.00)	0.265
ALT(U/L)	18.00 (11.00 - 34.00)	13.00 (9.00 - 18.00)	<0.001
AST(U/L)	23.00 (16.00 - 34.00)	17.00 (14.00 - 22.00)	<0.001
Albumin (g/L)	35.80 (33.10 - 37.90)	36.40 (32.50 - 39.00)	0.561
Anemia	23 (19.01%)	2 (1.47%)	<0.001
Creatinine (µmol/L)	51.10 (45.30 - 59.50)	52.00 (45.00 - 60.00)	0.940
FPG_32_36 (mmol/L)	4.85 (4.36 - 5.40)	5.18 (4.62 - 5.75)	0.002
LDH (U/L)	200.00 (173.00 - 225.00)	189.50 (163.00 - 208.25)	0.046
OGTT-0h (mmol/L)	5.16 (4.80 - 5.41)	5.13 (4.68 - 5.49)	0.769
OGTT-1h (mmol/L)	10.11 (8.95 - 11.11)	10.26 ± 1.81	0.389
OGTT-2h (mmol/L)	8.52 (7.46 - 9.50)	8.77 (8.00 - 9.46)	0.110
Total Bilirubin (µmol/L)	10.40 (6.30 - 12.60)	4.85 (3.60 - 6.90)	<0.001
Uric Acid (µmol/L)	371.00 (311.00 - 431.00)	352.45 ± 91.63	0.048
Educational Level			0.091
·Lower Secondary or Below	49 (40.50%)	41 (30.15%)	
·Tertiary or Above	49 (40.50%)	55 (40.44%)	
·Upper Secondary	23 (19.01%)	40 (29.41%)	
Cardiovascular Disease	11 (9.09%)	0 (0.00%)	0.001
Cesarean Scar Uterus	22 (18.18%)	34 (25.00%)	0.242
History of GDM	5 (4.13%)	0 (0.00%)	0.052
History of HDP	14 (11.57%)	0 (0.00%)	<0.001
Placenta Previa	1 (0.83%)	3 (2.21%)	0.699
Twin Pregnancy	10 (8.26%)	0 (0.00%)	0.002
Weight_24_27 (kg)	70.00 (63.00 - 77.60)	70.35 (63.00 - 79.00)	0.941
Weight_28_31 (kg)	73.00 (65.60 - 80.50)	71.00 (64.15 - 81.05)	0.504
Weight_32_36 (kg)	73.30 (66.70 - 81.30)	73.65 (66.38 - 83.00)	0.944

Dynamic variables, including systolic blood pressure (SBP) and diastolic blood pressure (DBP), maternal weight, and glucose measurements [fasting plasma glucose (FPG), oral glucose tolerance test (OGTT)], were collected longitudinally across specific gestational intervals (e.g., 8–15, 16–19, …, 32–36 weeks). Each is labeled in the format SBP_X_Y, DBP_X_Y, Weight_X_Y, or FPG_X_Y, where X and Y indicate the starting and ending gestational weeks (X+0 to Y+6). For example, SBP_32_36 denotes systolic blood pressure measured between 32+0 and 36+6 weeks of gestation, and Weight_24_27 refers to maternal weight during 24+0 to 27+6 weeks. OGTT-related variables follow the format OGTT-T, where T indicates the sampling time in hours (0h, 1h, or 2h) during the oral glucose tolerance test. Continuous variables are expressed as mean ± standard deviation (SD) when both groups were normally distributed, and as median (interquartile range [IQR]) otherwise. Between-group differences were assessed using the independent-samples t-test or Mann–Whitney U test as appropriate. Categorical variables are shown as n (%) and compared using the χ² test or Fisher’s exact test. Percentages are calculated within each cohort (A: n=121; B: n=136) and may not sum to 100% due to rounding. Blood pressure values are measured in mmHg with discrete increments, which may yield identical medians across time windows; the accompanying IQRs reflect the underlying distribution. Missing values were imputed using the MissForest algorithm. Cohorts: A = Sichuan Provincial People’s Hospital (development cohort); B = Tongji Hospital, Tongji Medical College, Huazhong University of Science and Technology (external validation cohort). OR, odds ratio; BMI, body mass index; IVF-ET, in vitro fertilization and embryo transfer; SBP, systolic blood pressure; DBP, diastolic blood pressure; ALT, alanine aminotransferase; AST, aspartate aminotransferase; FPG, fasting plasma glucose; LDH, lactate dehydrogenase; OGTT, oral glucose tolerance test; GDM, gestational diabetes mellitus; HDP, hypertensive disorders of pregnancy.

It can be concluded from **Table 2**, based on the results of univariate logistic regression analysis, that eight variables were found to be significantly associated with preterm birth (P < 0.05). Among them, albumin acted as a protective factor (odds ratio [OR] = 0.735, 95% confidence interval [CI]: 0.639–0.844, P < 0.001), indicating that higher albumin levels were associated with a lower risk of preterm birth. In contrast, elevated levels of LDH, systolic blood pressure (SBP_32_36 and SBP_28_31), diastolic blood pressure (DBP_32_36), Twin Pregnancy, Medication, and IVF-ET were identified as significant risk factors. For example, IVF-ET showed a strong positive association with preterm birth (OR = 5.160, 95% CI: 1.349 – 19.731, P = 0.016). Additionally, variables such as Total Bilirubin, ALT, and AST demonstrated potential associations with the outcome (P < 0.20), indicating potential predictive value. Given their potential predictive value, these variables were retained as candidate predictors for inclusion in the subsequent Elastic Net modeling process.

**Table 2:** Univariate logistic regression results.

Variable	P	OR (95% CI)
Age (years)	0.452	1.035 (0.946 - 1.132)
BMI (kg/m²)	0.928	1.004 (0.924 - 1.091)
Adverse Pregnancy History	0.584	1.500 (0.352 - 6.397)
Primiparity	0.273	1.664 (0.669 - 4.142)
IVF-ET	0.016	5.160 (1.349 - 19.731)
Medication	0.009	3.150 (1.329 - 7.467)
SBP_8_15 (mmHg)	0.650	0.993 (0.965 - 1.022)
SBP_16-19 (mmHg)	0.751	0.995 (0.967 - 1.025)
SBP_20_23 (mmHg)	0.878	1.003 (0.969 - 1.038)
SBP_24_27 (mmHg)	0.386	1.018 (0.977 - 1.061)
SBP_28_31 (mmHg)	0.043	1.040 (1.001 - 1.081)
SBP_32_36 (mmHg)	0.001	1.069 (1.027 - 1.113)
DBP_8_15 (mmHg)	0.905	0.998 (0.957 - 1.039)
DBP_16_19 (mmHg)	0.775	1.006 (0.968 - 1.045)
DBP_20_23 (mmHg)	0.639	1.010 (0.969 - 1.052)
DBP_24_27 (mmHg)	0.173	1.030 (0.987 - 1.074)
DBP_28_31 (mmHg)	0.080	1.043 (0.995 - 1.093)
DBP_32_36 (mmHg)	0.039	1.049 (1.003 - 1.098)
ALT (U/L)	0.086	1.005 (0.999 - 1.011)
AST (U/L)	0.112	1.011 (0.997 - 1.025)
Albumin (U/L)	<0.001	0.735 (0.639 - 0.844)
Anemia	0.558	1.349 (0.496 - 3.668)
Creatinine (µmol/L)	0.723	0.998 (0.984 - 1.011)
FPG_32_36 (mmol/L)	0.454	0.861 (0.583 - 1.273)
LDH (U/L)	<0.001	1.014 (1.006 - 1.023)
OGTT-0h (mmol/L)	0.363	1.321 (0.725 - 2.407)
OGTT-1h (mmol/L)	0.237	1.131 (0.922 - 1.386)
OGTT-2h (mmol/L)	0.217	1.128 (0.932 - 1.365)
Total Bilirubin (µmol/L)	0.079	1.090 (0.990 - 1.201)
Uric Acid (µmol/L)	0.375	1.002 (0.998 - 1.005)
Educational Level	0.356	0.806 (0.510 - 1.274)
Cardiovascular Disease	0.557	0.621 (0.127 - 3.043)
Cesarean Scar Uterus	0.206	1.888 (0.704 - 5.061)
History of GDM	0.770	0.717 (0.077 - 6.668)
History of HDP	0.788	1.185 (0.343 - 4.091)
Placenta Previa	1.000	5.192e+14 (0.000-inf)
Twin Pregnancy	0.003	8.458 (2.032 - 35.205)
Weight_24_27 (kg)	0.973	0.999 (0.968 - 1.032)
Weight_28_31 (kg)	0.693	1.006 (0.976 - 1.038)
Weight_32_36 (kg)	0.583	1.009 (0.978 - 1.040)

Note: Dynamic variables, including systolic blood pressure (SBP) and diastolic blood pressure (DBP), maternal weight, and glucose measurements [fasting plasma glucose (FPG), oral glucose tolerance test (OGTT)], were collected longitudinally across specific gestational intervals (e.g., 8–15, 16–19, …, 32–36 weeks). Each is labeled in the format SBP_X_Y, DBP_X_Y, Weight_X_Y, or FPG_X_Y, where X and Y indicate the starting and ending gestational weeks (X+0 to Y+6). For example, SBP_32_36 denotes systolic blood pressure measured between 32+0 and 36+6 weeks of gestation, and Weight_24_27 refers to maternal weight during 24+0 to 27+6 weeks. OGTT-related variables follow the format OGTT-T, where T indicates the sampling time in hours (0h, 1h, or 2h) during the oral glucose tolerance test. OR, odds ratio; 95% CI, 95% confidence interval; BMI, body mass index; IVF-ET, in vitro fertilization and embryo transfer; SBP, systolic blood pressure; DBP, diastolic blood pressure; ALT, alanine aminotransferase; AST, aspartate aminotransferase; FPG, fasting plasma glucose; LDH, lactate dehydrogenase; OGTT, oral glucose tolerance test; GDM, gestational diabetes mellitus; HDP, hypertensive disorders of pregnancy.

**Figure 1:**
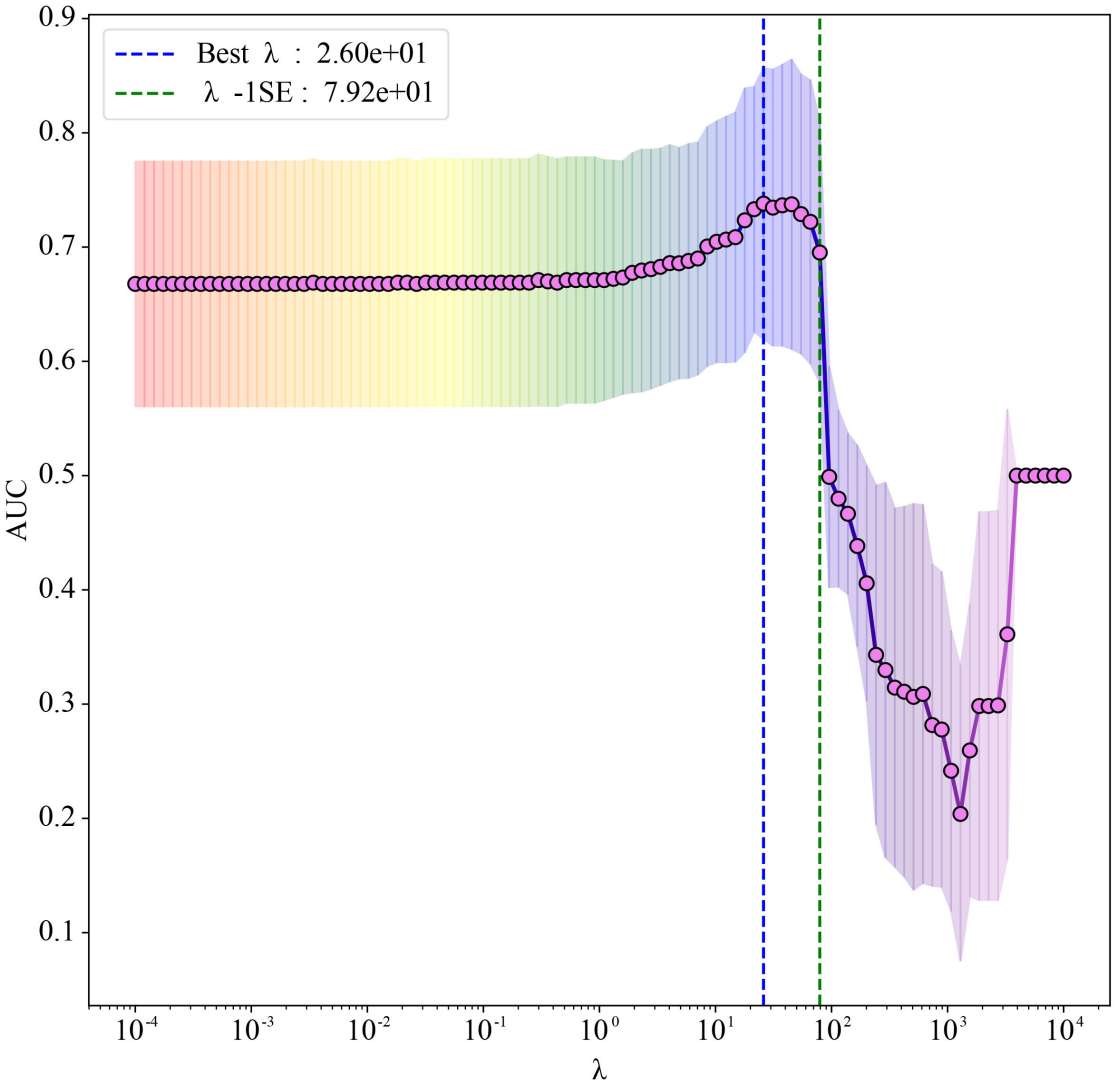
Elastic Net regression coefficient path plot. The abscissa shows λ on a logarithmic scale (larger λ indicates stronger regularization); the ordinate reports the mean area under the receiver operating characteristic (ROC) curve (AUC). Red markers joined by a line represent the mean AUC obtained from three-fold cross-validation at each λ. Grey vertical bars denote ±1 standard error (SE), reflecting variability across folds. The blue dashed line marks the Best λ (λmin = 26), which yields the best mean AUC, whereas the green dashed line marks λ−1SE (79.2); selecting λ−1SE gives a more parsimonious model at negligible loss of discrimination.

Based on univariate analysis (P < 0.20), thirteen candidate predictors were included in the Elastic Net regression model. To select the optimal features, Elastic Net regression was performed using three-fold cross-validation, and the minimum mean squared error criterion (λ-min) was applied. As a result, five predictors—ALT, AST, Albumin, LDH, and SBP_32_36—were ultimately retained for model development (the antihypertensive medication variable, although initially considered among the candidates, was not retained). The coefficient path is illustrated in **Figure 1**. Elastic Net regression retained a set of predictors that were most informative for preterm birth risk. The selected predictors, together with their regression coefficients and direction of association, are summarized in **Table 3**. These coefficients reflect the relative importance of each predictor within the penalized regression framework, where larger absolute values indicate stronger contributions to the model.

**Table 3:** Retained predictors and coefficients from elastic net regression.

Predictor	Coefficient	Relationship
ALT (U/L)	0.123	Positive
AST (U/L)	-0.087	Negative
Albumin (U/L)	-0.145	Negative
LDH (U/L)	0.256	Positive
SBP_32_36 (mmHg)	0.178	Positive

Coefficients are standardized and retained to three decimal places. Positive coefficients indicate increased risk of preterm birth, while negative coefficients indicate a protective association. SBP_32_36 refers to systolic blood pressure measured between 32+0 and 36+6 weeks of gestation. ALT, alanine aminotransferase; AST, aspartate aminotransferase; LDH, lactate dehydrogenase; SBP, systolic blood pressure.

### 3.2 Model performance

**Figure 2:**
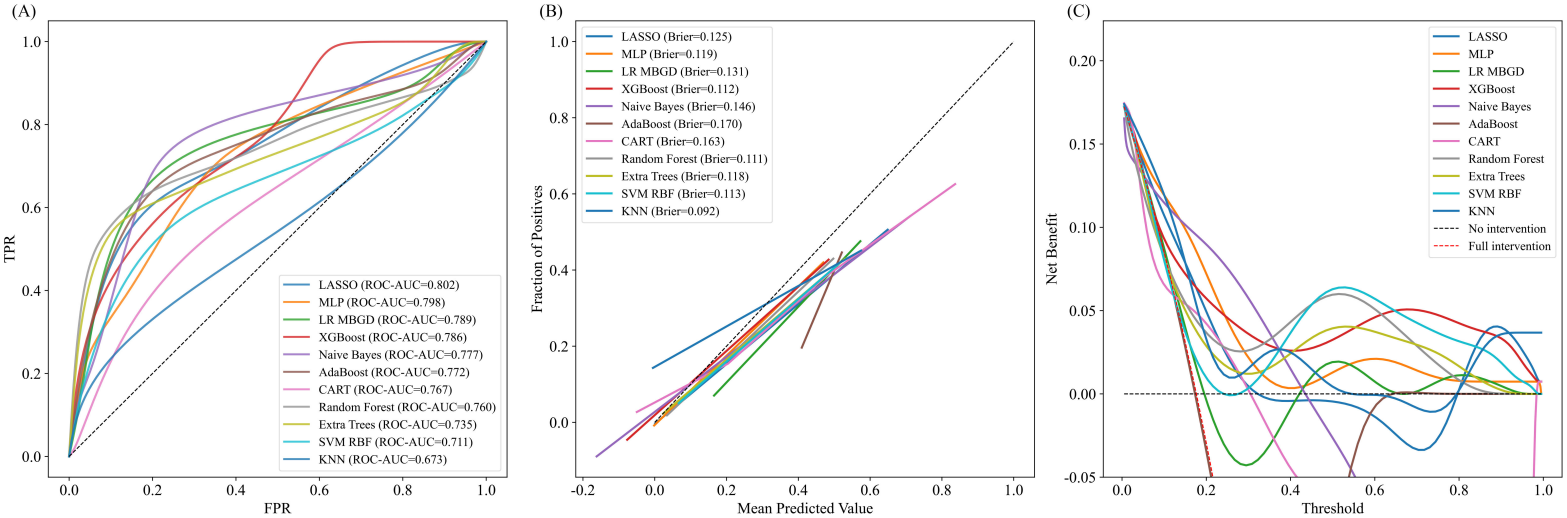
Comprehensive evaluation of predictive models in the external validation cohort. **(A)** Receiver operating characteristic (ROC) curves of predictive models in the external validation cohort. X-axis: False positive rate (FPR); Y-axis: True positive rate (TPR). The curves show each model’s ability to discriminate between positive and negative outcomes. A curve closer to the top-left corner indicates better performance. The area under the curve (AUC) for each model is reported in the legend. **(B)** Calibration curves of different models. X-axis: Mean Predicted Value (probability of positive outcome); Y-axis: Fraction of Positives (observed outcome rate). The dashed diagonal line represents perfect calibration. The closer a model’s curve is to this line, the better its predicted probabilities align with observed outcomes. Each model’s Brier score is shown in the legend, with lower scores indicating more accurate calibration. **(C)** Decision curve analysis (DCA) of different models. X-axis: Threshold probability; Y-axis: Net benefit. The curves assess the clinical utility of each model across a range of threshold probabilities. Dashed lines represent the “no intervention” (black) and “full intervention” (red) strategies. Curves above these lines indicate greater net benefit. FPR, False Positive Rate; TPR, True Positive Rate; LASSO, Least Absolute Shrinkage and Selection Operator; MLP, Multilayer Perceptron; LR MBGD, Logistic Regression trained with Mini-Batch Gradient Descent; XGBoost, Extreme Gradient Boosting; AdaBoost, Adaptive Boosting; CART, Classification and Regression Tree; Extra Trees, Extremely Randomized Trees; SVM RBF, Support Vector Machine with Radial Basis Function kernel; KNN, K-Nearest Neighbors.

Using these five variables, we constructed predictive models with Logistic Regression, Random Forest (RF), NB, Support Vector Machine (SVM), and other algorithms. All models were tuned and trained using three-fold cross-validation in the development cohort, and SMOTE was applied to address class imbalance before model training. In the external validation cohort, the LASSO model achieved the highest discrimination performance with an AUC of 0.802, followed closely by the Multilayer Perceptron (MLP, AUC = 0.798) and Logistic Regression with mini-batch gradient descent (LR MBGD, AUC = 0.789). The AUCs of XGBoost, NB, and AdaBoost models were 0.786, 0.777, and 0.772, respectively, indicating moderate discriminative ability. Notably, the Naive Bayes model demonstrated reasonable discrimination (AUC = 0.777), ranking in the upper-middle range among all tested algorithms. Although its AUC was slightly lower than those of LASSO, MLP, and some relatively well-performing ensemble models such as XGBoost, it outperformed several classical approaches, such as Classification and Regression Tree (CART, AUC = 0.767), Random Forest (AUC = 0.760), and SVM (AUC = 0.711). In contrast, the K-Nearest Neighbor (KNN, AUC = 0.673) model showed the weakest discriminative power. These findings suggest that the LASSO model provided the best discriminatory power for identifying preterm birth risk; however, notably, the Naive Bayes model also demonstrated acceptable and stable discrimination, thereby justifying its inclusion in further evaluation. **Figure 2A** presents the ROC curves along with the corresponding AUC values for the models in the external validation cohort. Detailed performance metrics, including accuracy, F1 score, sensitivity, specificity, positive predictive value, and negative predictive value for each model in the development and external validation cohorts, are presented in **Supplementary Table S1**.

The calibration performance of all models was evaluated using calibration curves and Brier scores in the external validation cohort (**Figure 2B**). Models with curves closer to the diagonal line demonstrated better alignment between predicted probabilities and observed outcomes. Among the tested models, K-Nearest Neighbor exhibited the best calibration performance, with the lowest Brier score of 0.092, indicating high prediction reliability. Other well-calibrated models included Random Forest (0.111), XGBoost (0.112), and SVM RBF (0.113).

Although the Naive Bayes model demonstrated acceptable discriminatory ability, its calibration performance was suboptimal, with a Brier score of 0.146. The calibration curve deviated upward from the diagonal in the low-to mid-probability range, indicating a tendency to underestimate the actual risk. Similarly, AdaBoost (0.170) and CART (0.163) displayed suboptimal calibration, with predicted probabilities deviating more from actual event rates.

### 3.3 Clinical utility of the model

**Figure 3:**
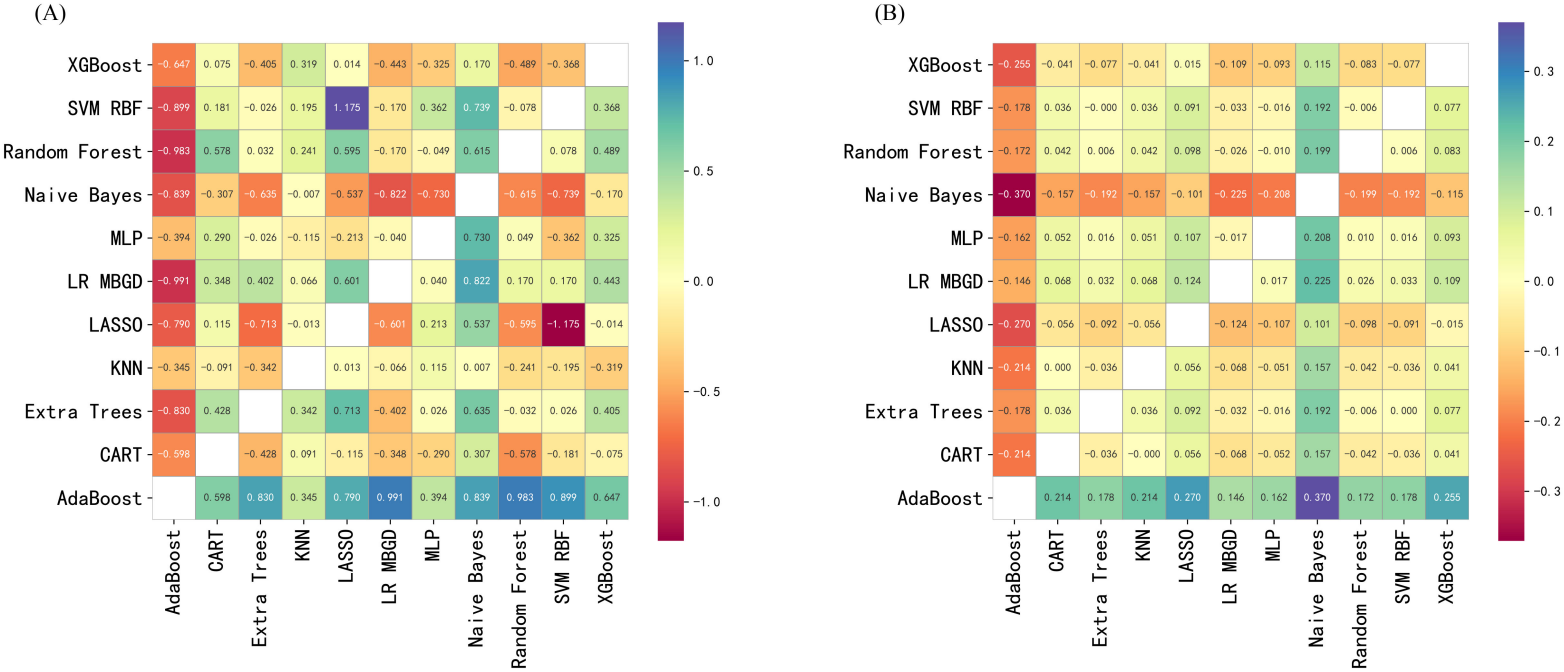
Pairwise reclassification performance of predictive models in the external validation cohort. **(A)** Pairwise net reclassification improvement (NRI) comparison between models in the external validation cohort. X-axis: Comparator model; Y-axis: Reference model. Each cell displays the NRI value comparing the model on the y-axis with that on the x-axis. Positive values represent better reclassification by the x-axis model. Cooler colors indicate performance gain; warmer colors indicate performance loss. **(B)** Pairwise integrated discrimination improvement (IDI) comparison between models in the external validation cohort. X-axis: Comparator model; Y-axis: Reference model. Each cell displays the IDI value when comparing the model on the y-axis with the model on the x-axis. Positive values indicate that the x-axis model outperforms the y-axis model in terms of discrimination. Color intensity indicates the magnitude and direction of improvement. XGBoost, Extreme Gradient Boosting; SVM RBF, Support Vector Machine with Radial Basis Function kernel; MLP, Multilayer Perceptron; LR MBGD, Logistic Regression trained with Mini-Batch Gradient Descent; LASSO, Least Absolute Shrinkage and Selection Operator; KNN, K-Nearest Neighbors; Extra Trees, Extremely Randomized Trees; CART, Classification and Regression Tree; AdaBoost, Adaptive Boosting.

DCA was performed to evaluate the clinical utility of each model across a range of threshold probabilities in the external validation cohort (**Figure 2C**). Overall, XGBoost, Random Forest, and MLP models yielded the highest net benefit across the clinically relevant threshold range of 0.2 – 0.6, suggesting superior performance in guiding clinical decision-making. In contrast, models such as CART and AdaBoost exhibited consistently lower or even negative net benefit values, particularly in mid-to-high threshold ranges, indicating limited clinical value. Naive Bayes demonstrated modest net benefit in the low-threshold range (below 0.3), suggesting limited but potentially useful clinical utility for early risk screening. These findings suggest that while ensemble and deep learning models offer greater potential for risk-based intervention strategies, Naive Bayes still retains some value in early-risk screening contexts. To identify the most clinically useful model, we further compared all candidate algorithms using reclassification metrics, including NRI and IDI. As illustrated in the heatmaps (**Figures 3A, B**), the Naive Bayes model outperformed most other candidates in both Test-NRI and Test-IDI, demonstrating the strongest ability to improve risk stratification across clinically relevant thresholds. Although its AUC and calibration performance were only moderate, these reclassification advantages, combined with adequate discriminatory ability, led to its selection as the final model for individual-level prediction and clinical interpretation.

**Figure 4:**
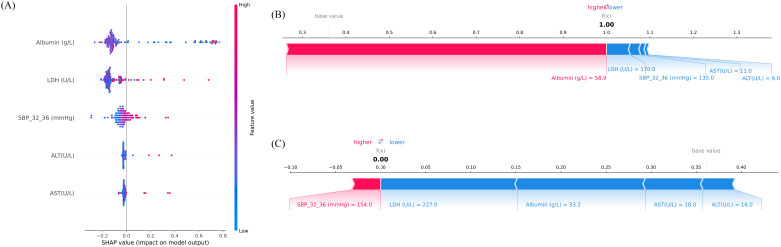
Interpretation of model predictions for preterm birth risk using Shapley additive explanations (SHAP) analysis. **(A)** SHAP summary plot illustrating the importance and direction of influence of the top five features included in the final model. The x-axis represents SHAP values, which indicate the magnitude and direction of each feature’s contribution to the predicted risk (positive = higher predicted risk, negative = lower predicted risk). The y-axis lists the features ranked by mean absolute SHAP value. The color bar encodes the feature’s original value (red = higher value, blue = lower value). Each point corresponds to an individual sample. **(B)** SHAP force plot for an individual case with high predicted risk (predicted probability = 1.00). Red segments indicate predictors increasing risk, while blue segments indicate predictors reducing risk. **(C)** SHAP force plot for an individual case with low predicted risk (predicted probability = 0.00). Blue segments dominate, indicating overall protective contributions. The balance between positive (red) and negative (blue) contributions determines the final prediction output. SBP_32_36, systolic blood pressure measured between 32+0 and 36+6 weeks of gestation. AST, aspartate aminotransferase; ALT, alanine aminotransferase; LDH, lactate dehydrogenase; SBP, systolic blood pressure.

To further explain the contribution and directionality of each predictor, SHAP analysis was applied. A SHAP summary plot was generated to visualize the global feature importance across all samples (**Figure 4A**). Each point represents an individual case, with color indicating the feature value (red for high, blue for low), and horizontal position denoting the SHAP value, which reflects the magnitude and direction of impact on the model’s output. Among the five selected predictors, Albumin, LDH, and SBP at 32 – 36 weeks exhibited the strongest influence, confirming their central role in risk prediction. Notably, lower albumin and higher LDH levels were associated with increased predicted risk, consistent with known clinical mechanisms.

To illustrate the model’s behavior at the individual level, SHAP force plots were generated for two representative patients (**Figures 4B, C**). **Figure 4B** depicts a high-risk case with a predicted preterm birth probability of 1.00. In this individual, elevated albumin levels unexpectedly emerged as the dominant positive contributor, illustrating case-specific variability in SHAP explanations. In contrast, **Figure 4C** presents a low-risk individual, where negative contributions from LDH, Albumin, AST, and ALT outweighed a small positive effect of SBP_32_36, resulting in a near-zero predicted. These visualizations reflect the model’s nuanced understanding of inter-feature dependencies and support its utility in personalized risk assessment. 

### 3.4 Sensitivity analysis

**Figure 5:**
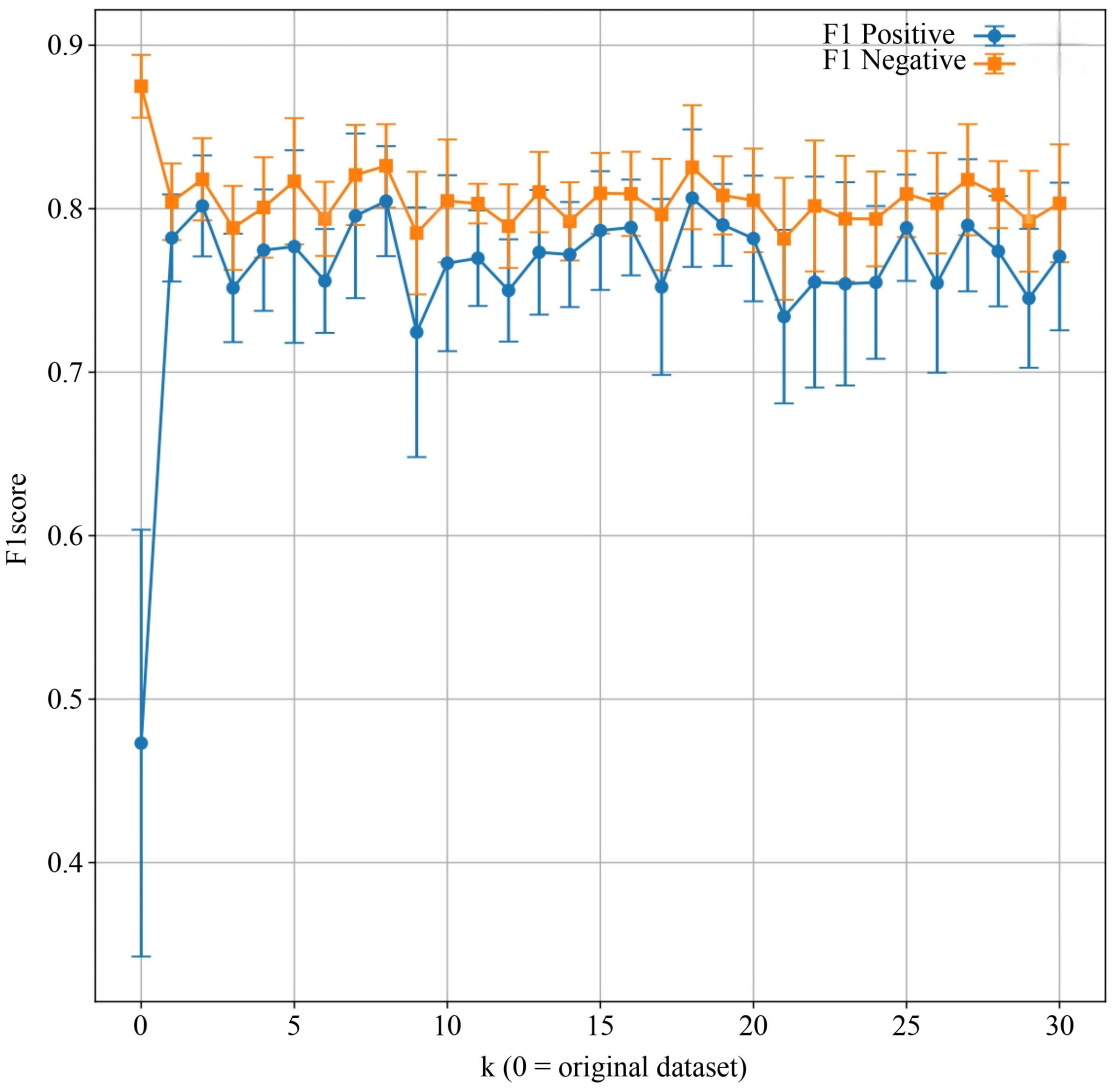
Comparison of model F1 scores before and after applying the Synthetic Minority Over-sampling Technique (SMOTE). X-axis: Number of nearest neighbors parameter k in SVMSMOTE (k = 0 indicates the original dataset); Y-axis: F1 score. Each point represents the mean F1 score across cross-validation folds for positive and negative classes, with error bars indicating standard deviation. The blue line tracks F1 scores for the positive class, while the orange line represents the negative class.

**Figure 5** illustrates the effect of varying the number of neighbors (*k*) in SVMSMOTE on F1 scores for both positive and negative classes. The model exhibited improved and more stable performance for the minority class when *k* ≥ 5. Performance trends help identify the optimal *k* for balanced classification. Considering discrimination, calibration, reclassification performance, individual interpretability, and clinical net benefit, the Naive Bayes model was identified as the optimal preterm birth risk prediction model in this study, demonstrating good external generalizability and practical application prospects.

## 4 Discussion

Preterm birth remains a leading contributor to perinatal morbidity and mortality (4), and prediction continues to be particularly challenging in women with comorbid GDM and HDP. Population-based data demonstrate markedly higher risks in GDM–HDP pregnancies compared to either condition alone (21). Prior prediction models have typically addressed only GDM or HDP in isolation, reporting moderate discrimination and limited clinical utility (22). To our knowledge, no existing model specifically targets the combined GDM–HDP population, despite its elevated baseline risk. By focusing on this high-risk group and performing external validation, the present study adds important evidence to this limited domain and proposes a clinically interpretable model that balances predictive performance with translational feasibility. Recent advances in AI and interpretable machine learning further support the feasibility of deploying such models in obstetric practice (23).

Modern ensemble algorithms such as Random Forest and XGBoost have frequently been highlighted for superior discrimination in obstetric prediction (24, 25). However, their complexity and limited interpretability have restricted clinical translation, echoing concerns raised in other obstetric risk prediction tasks, including postpartum hemorrhage (25, 26). In our cohorts, LASSO achieved the highest AUC, and ensembles demonstrated favorable net benefit in decision-curve analyses (27, 28). However, the Naive Bayes (NB) model, while showing a slightly lower AUC (~0.78), achieved superior reclassification (NRI/IDI) and maintained competitive calibration. More importantly, its transparent probabilistic framework provides directly interpretable risk probabilities, which we considered a decisive advantage for practical obstetric risk counseling. This interpretability, combined with the model’s simplicity and routine clinical availability of its predictors, underscores its translational potential in obstetric practice despite the modest AUC. Given the modest sample size and the comparison of multiple algorithms, we acknowledge the potential risk of overfitting and the *post hoc* nature of model selection. This deliberate trade-off underscores that marginal improvements in AUC may be less clinically meaningful than ensuring interpretability and usability in high-risk care pathways. Accordingly, NB was prioritized for primary reporting, as its simplicity and interpretability enhance translational potential and reduce the risk of overfitting in modest obstetric datasets, aligning with prior calls for clinically explainable prediction models (23).

Beyond model performance, the retained predictors also reflect strong biological plausibility. Elevated hepatic enzymes (ALT, AST) have consistently been associated with preeclampsia severity and adverse maternal outcomes (29–31), and our findings extend this evidence by demonstrating their predictive value in a multimorbidity cohort where hepatocellular injury and systemic inflammation may converge with metabolic stress from GDM (32). Albumin, although underused in prior prediction models, has been linked to endothelial dysfunction, maternal malnutrition, and fluid imbalance (32), and its inclusion here emphasizes the interplay between hepatic reserve and vascular integrity in dual-risk pregnancies. Similarly, LDH has long been reported as a marker of cellular injury and oxidative stress in severe HDP (33). While earlier studies positioned LDH primarily as a late indicator of disease severity, our results suggest a broader role in the GDM–HDP setting, integrating systemic hypoxic stress with metabolic dysregulation to capture maternal–placental strain more comprehensively. Compared with organ-specific enzymes, LDH reflects systemic cascades, consistent with recent arguments on systemic predictors of pregnancy complications (35).

Hemodynamic adaptation in late gestation further reinforces this multi-domain perspective. Blood pressure has long been recognized as central to pregnancy outcomes (35), yet most prior prediction models have focused on early-pregnancy measures for anticipatory stratification (36). Our identification of systolic blood pressure at 32 – 36 weeks (SBP_32_36) as an independent predictor highlights the prognostic significance of late-gestational dynamics. Elevated SBP in this window likely reflects cumulative vascular burden and declining compensatory capacity, suggesting that temporal patterns of blood pressure provide additional prognostic information. By incorporating such longitudinal measures, our approach moves prediction closer to real-time surveillance, consistent with precision obstetric care initiatives (37).

Taken together, these findings both corroborate and extend existing literature. They confirm the roles of hepatic enzymes (29–31), LDH (34), and blood pressure trajectories (35), but in a broader context that integrates multimorbidity rather than single conditions. By situating individual predictors within the dual-risk framework of GDM–HDP, our study highlights not only their continued relevance but also new dimensions, such as the overlooked predictive value of late-gestational SBP. Divergences from prior reports likely reflect differences in study populations, sample sizes, and timing of data collection, but they also underscore the distinctive pathophysiology of multimorbidity. Thus, our study provides a more integrated and clinically relevant framework for preterm birth prediction. Importantly, the interpretability of the model strengthens its applicability in clinical practice. SHAP-based visualizations at both population and individual levels allowed us to bridge predictive accuracy with explainability (38), a recognized barrier to adoption of Artificial Intelligence in obstetrics. SHAP summary plots consistently ranked LDH, albumin, and SBP_32_36 among the most influential predictors, reinforcing their biological plausibility and echoing recent applications of SHAP in preeclampsia and postpartum hemorrhage models. Notably, SHAP plots also suggested a tendency to underestimate risk among the lowest-risk strata, which may reflect data imbalance and calibration limitations. In parallel, patient-level SHAP force plots decomposed individual risk profiles into positive and negative contributions, offering a pathway to targeted monitoring and intervention. In addition, we leveraged the intrinsic transparency of the Naive Bayes model itself, whose conditional probabilities directly reflect the contribution of each predictor to the overall risk. This NB-specific interpretability complements the SHAP explanations, providing clinicians with a more intuitive understanding of risk attribution. Although SHAP may introduce approximation errors and relies on assumptions of feature independence, these limitations are mitigated within the simple probabilistic structure of NB.

Clinical relevance further underscores the potential utility of our model. Because all five predictors are routinely measured in antenatal care, the model can be seamlessly applied in real-world workflows without additional testing burden. In particular, SBP measured at 32 – 36 weeks provides a practical window to inform delivery planning and closer surveillance. While the model may add limited value in cases of overtly severe GDM or HDP, it offers important guidance in borderline or ambiguous presentations, helping clinicians to stratify risk more objectively. Moreover, by integrating multiple predictors, the model can reveal hidden risk profiles that may not be apparent when considering single variables in isolation. This potential to enhance workflow efficiency and provide early warnings highlights its clinical value. However, the model should be viewed as a decision-support tool that may aid in risk stratification and clinical management, rather than one that directly improves outcomes.

To evaluate real-world robustness, we also assessed the impact of class imbalance. The rarity of adverse outcomes often limits model performance, and prior work has noted the instability of ensemble methods under imbalance (39). In our analyses, the NB model maintained stable calibration and discrimination following SMOTE while improving recall. Sensitivity analyses further indicated that SMOTE improved recall and balanced accuracy without substantially altering the AUC or calibration, suggesting that the oversampling procedure effectively mitigated class imbalance without introducing significant bias. Comparable trends were observed without SMOTE, albeit with slightly reduced discrimination, supporting the robustness of the findings. This indicates that NB, combined with oversampling, may be particularly suitable for obstetric applications where event rates are low and interpretability is essential.

Finally, strengths and limitations warrant consideration. This is, to our knowledge, the first study to develop and externally validate a preterm birth prediction model specifically for women with comorbid GDM and HDP. Strengths include the use of dual-center data to enhance generalizability; the integration of five clinically accessible, biologically coherent predictors spanning complementary physiological domains; variable selection through Elastic Net to address multicollinearity, followed by a robust yet simple NB classifier; and a comprehensive multi-metric evaluation covering discrimination, calibration, clinical utility, reclassification, and interpretability. Limitations include its retrospective design, the relatively small sample size (particularly in the external validation cohort), potential selection bias, the absence of intervention-based validation, and the possibility that the findings may not be directly generalizable to populations outside China. In addition, we recognize the possibility of overfitting due to modest sample size and the *post hoc* nature of model choice. Moreover, some clinically relevant domains such as psychosocial, nutritional, or imaging biomarkers were not included. Medication variables were also coarse, with antihypertensive use captured only as a binary yes/no indicator and glucose-lowering therapies not systematically recorded, which may limit interpretability. Future research should pursue prospective and multicenter validation, explore integration into clinical workflows via electronic health records or mobile/desktop applications, and evaluate whether model-guided interventions can improve maternal and neonatal outcomes.

## 5 Conclusions

In this study, we developed and externally validated multiple predictive models to assess the risk of preterm birth in pregnancies complicated by both GDM and HDP. Among the evaluated algorithms, the Naive Bayes classifier demonstrated the most favorable balance across discrimination, reclassification, interpretability, and robustness, and was ultimately selected as the optimal model for clinical application. Through Elastic Net regression, five physiologically meaningful predictors—ALT, AST, albumin, LDH, and systolic blood pressure at 32 – 36 weeks—were identified and incorporated into model development. These variables capture distinct domains relevant to preterm labor pathophysiology, including hepatic dysfunction, systemic inflammation, vascular insufficiency, and hemodynamic instability. To enhance transparency and clinical utility, SHAP-based interpretation techniques were applied at both the global and individual levels. Summary plots highlighted the dominant predictors at the population level, while force plots provided case-specific insights into individualized risk contributions. Additionally, SMOTE-based sensitivity analysis confirmed the Naive Bayes model’s robustness under class imbalance, further supporting its generalizability and deployment potential.

The proposed Naive Bayes model may assist clinicians in early identification and personalized risk management of high-risk pregnancies affected by GDM and HDP, and represents a step toward the implementation of transparent, evidence-based decision support in obstetric practice. Future studies should aim to validate this model in larger, multicenter cohorts and explore its integration into real-time clinical decision support systems.

## Data Availability

The original contributions presented in the study are included in the article/**Supplementary Material**. Further inquiries can be directed to the corresponding authors.
