# Supplementary material for: An explainable machine learning model for predicting preterm birth in pregnant women with gestational diabetes mellitus and hypertensive disorders of pregnancy: development and external validation
Source: Front Endocrinol (Lausanne). 2025 Nov 18;16:1665935. doi: 10.3389/fendo.2025.1665935 (PMC12668920; doi:10.3389/fendo.2025.1665935)
Supplement: Supplementary file 1 [file Table1.docx]

| Model | Dataset | AUC（95%Cl） | Accuracy（95%Cl） | F1 score（95%Cl） | Sensitivity（95%Cl） | Specificity（95%Cl） | Youden Index（95%CI） | PPV（95%Cl） | NPV（95%Cl） | Threshold |
| --- | --- | --- | --- | --- | --- | --- | --- | --- | --- | --- |
| AdaBoost | Training | 0.996 (0.988-1.000) | 0.975 (0.942-1.000) | 0.952 (0.889-1.000) | 0.968 (0.880-1.000) | 0.978 (0.943-1.000) | 0.946 (0.861-1.000) | 0.938 (0.846-1.000) | 0.989 (0.964-1.000) | 0.497 |
| CART | Training | 0.955 (0.920-0.981) | 0.909 (0.860-0.951) | 0.800 (0.667-0.905) | 0.710 (0.552-0.857) | 0.978 (0.943-1.000) | 0.687 (0.508-0.839) | 0.917 (0.783-1.000) | 0.907 (0.845-0.958) | 0.875 |
| Extra Trees | Training | 0.968 (0.919-1.000) | 0.983 (0.959-1.000) | 0.967 (0.912-1.000) | 0.935 (0.839-1.000) | 1.000 (1.000-1.000) | 0.935 (0.839-1.000) | 1.000 (1.000-1.000) | 0.978 (0.944-1.000) | 1.000 |
| KNN | Training | 0.934 (0.894-0.967) | 0.851 (0.785-0.909) | 0.727 (0.596-0.842) | 0.774 (0.613-0.914) | 0.878 (0.806-0.942) | 0.652 (0.488-0.808) | 0.686 (0.526-0.833) | 0.919 (0.854-0.967) | 0.750 |
| LASSO | Training | 0.810 (0.691-0.904) | 0.835 (0.769-0.893) | 0.667 (0.528-0.794) | 0.645 (0.469-0.808) | 0.900 (0.838-0.955) | 0.545 (0.361-0.709) | 0.690 (0.500-0.862) | 0.880 (0.813-0.942) | 0.559 |
| LightGBM | Training | 0.971 (0.923-1.000) | 0.983 (0.959-1.000) | 0.968 (0.915-1.000) | 0.968 (0.885-1.000) | 0.989 (0.964-1.000) | 0.957 (0.874-1.000) | 0.968 (0.892-1.000) | 0.989 (0.966-1.000) | 0.884 |
| MLP | Training | 0.891 (0.821-0.950) | 0.893 (0.835-0.942) | 0.755 (0.596-0.863) | 0.645 (0.467-0.818) | 0.978 (0.942-1.000) | 0.623 (0.450-0.779) | 0.909 (0.778-1.000) | 0.889 (0.825-0.949) | 0.520 |
| Naive Bayes | Training | 0.934 (0.890-0.972) | 0.868 (0.802-0.926) | 0.742 (0.600-0.853) | 0.742 (0.583-0.889) | 0.911 (0.848-0.965) | 0.653 (0.473-0.811) | 0.742 (0.567-0.886) | 0.911 (0.849-0.959) | 0.353 |
| Random Forest | Training | 0.991 (0.977-1.000) | 0.983 (0.959-1.000) | 0.968 (0.915-1.000) | 0.968 (0.889-1.000) | 0.989 (0.964-1.000) | 0.957 (0.875-1.000) | 0.968 (0.885-1.000) | 0.989 (0.965-1.000) | 0.629 |
| SVM RBF | Training | 0.861 (0.758-0.947) | 0.876 (0.810-0.934) | 0.746 (0.610-0.857) | 0.710 (0.545-0.867) | 0.933 (0.875-0.978) | 0.643 (0.476-0.810) | 0.786 (0.625-0.933) | 0.903 (0.842-0.959) | 0.429 |
| XGBoost | Training | 0.964 (0.911-0.997) | 0.967 (0.934-0.992) | 0.935 (0.862-0.987) | 0.935 (0.838-1.000) | 0.978 (0.945-1.000) | 0.913 (0.805-0.990) | 0.935 (0.840-1.000) | 0.978 (0.946-1.000) | 0.655 |
| AdaBoost | Test | 0.772 (0.631-0.888) | 0.868 (0.809-0.919) | 0.625 (0.444-0.773) | 0.625 (0.429-0.818) | 0.920 (0.862-0.971) | 0.545 (0.325-0.740) | 0.625 (0.421-0.824) | 0.920 (0.864-0.964) | 0.497 |
| CART | Test | 0.767 (0.667-0.860) | 0.846 (0.787-0.904) | 0.571 (0.407-0.720) | 0.583 (0.389-0.786) | 0.902 (0.847-0.955) | 0.485 (0.268-0.688) | 0.560 (0.360-0.750) | 0.910 (0.852-0.955) | 0.548 |
| Extra Trees | Test | 0.735 (0.594-0.879) | 0.860 (0.801-0.919) | 0.578 (0.390-0.731) | 0.542 (0.333-0.760) | 0.929 (0.882-0.973) | 0.470 (0.255-0.675) | 0.619 (0.407-0.818) | 0.904 (0.853-0.951) | 0.469 |
| KNN | Test | 0.673 (0.546-0.788) | 0.838 (0.779-0.897) | 0.522 (0.300-0.694) | 0.500 (0.292-0.708) | 0.911 (0.856-0.958) | 0.411 (0.216-0.616) | 0.545 (0.333-0.750) | 0.895 (0.830-0.948) | 0.450 |
| LASSO | Test | 0.802 (0.712-0.892) | 0.860 (0.794-0.919) | 0.578 (0.375-0.744) | 0.542 (0.346-0.741) | 0.929 (0.878-0.973) | 0.470 (0.250-0.668) | 0.619 (0.391-0.824) | 0.904 (0.847-0.957) | 0.614 |
| LR MBGD | Test | 0.789 (0.657-0.906) | 0.868 (0.809-0.919) | 0.625 (0.444-0.778) | 0.625 (0.423-0.826) | 0.920 (0.868-0.965) | 0.545 (0.327-0.749) | 0.625 (0.429-0.800) | 0.920 (0.867-0.965) | 0.518 |
| MLP | Test | 0.798 (0.709-0.872) | 0.757 (0.684-0.824) | 0.459 (0.298-0.600) | 0.583 (0.370-0.773) | 0.795 (0.719-0.868) | 0.378 (0.143-0.584) | 0.378 (0.214-0.545) | 0.899 (0.833-0.953) | 0.345 |
| Naive Bayes | Test | 0.777 (0.645-0.887) | 0.801 (0.735-0.860) | 0.585 (0.435-0.713) | 0.792 (0.621-0.955) | 0.804 (0.729-0.877) | 0.595 (0.404-0.754) | 0.463 (0.300-0.617) | 0.947 (0.900-0.989) | 0.234 |
| Random Forest | Test | 0.760 (0.609-0.891) | 0.882 (0.824-0.934) | 0.619 (0.432-0.769) | 0.542 (0.333-0.739) | 0.955 (0.915-0.991) | 0.497 (0.284-0.706) | 0.722 (0.500-0.917) | 0.907 (0.856-0.955) | 0.464 |
| SVM RBF | Test | 0.711 (0.557-0.849) | 0.860 (0.801-0.919) | 0.596 (0.419-0.741) | 0.583 (0.381-0.762) | 0.920 (0.864-0.964) | 0.503 (0.294-0.703) | 0.609 (0.389-0.812) | 0.912 (0.859-0.957) | 0.428 |
| XGBoost | Test | 0.786 (0.677-0.873) | 0.860 (0.801-0.919) | 0.537 (0.333-0.700) | 0.458 (0.261-0.667) | 0.946 (0.901-0.983) | 0.405 (0.205-0.610) | 0.647 (0.400-0.882) | 0.891 (0.830-0.942) | 0.491 |

Abbreviations: Training = development cohort; Test = external validation cohort. AUC, area under the receiver operating characteristic (ROC) curve; 95%CI, 95%confidence interval; PPV, positive predictive value; NPV, negative predictive value; AdaBoost, Adaptive Boosting; CART, Classification and Regression Tree; Extra Trees, Extremely Randomized Trees; KNN, K-Nearest Neighbor; LASSO, Least Absolute Shrinkage and Selection Operator; LightGBM, Light Gradient Boosting Machine; MLP, Multilayer Perceptron; SVM RBF, Support Vector Machine with radial basis function kernel; XGBoost, Extreme Gradient Boosting.
